# Foundation model for cancer imaging biomarkers

**DOI:** 10.1038/s42256-024-00807-9

**Published:** 2024-03-15

**Authors:** Suraj Pai, Dennis Bontempi, Ibrahim Hadzic, Vasco Prudente, Mateo Sokač, Tafadzwa L. Chaunzwa, Simon Bernatz, Ahmed Hosny, Raymond H. Mak, Nicolai J. Birkbak, Hugo J. W. L. Aerts

**Affiliations:** 1 Artificial Intelligence in Medicine (AIM) Program, Mass General Brigham, Harvard Medical School, Harvard Institutes of Medicine, Boston, MA USA; 2 Radiology and Nuclear Medicine, CARIM and GROW, Maastricht University, Maastricht, the Netherlands; 3 Department of Radiation Oncology, Brigham and Women’s Hospital, Dana-Farber Cancer Institute, Harvard Medical School, Boston, MA USA; 4 Department of Molecular Medicine, Aarhus University Hospital, Aarhus, Denmark; 5 Department of Clinical Medicine, Aarhus University, Aarhus, Denmark; 6 Department of Radiology, Brigham and Women’s Hospital, Dana-Farber Cancer Institute, Harvard Medical School, Boston, MA USA

**Keywords:** Biomarkers, Tumour biomarkers, Cancer imaging

## Abstract

Foundation models in deep learning are characterized by a single large-scale model trained on vast amounts of data serving as the foundation for various downstream tasks. Foundation models are generally trained using self-supervised learning and excel in reducing the demand for training samples in downstream applications. This is especially important in medicine, where large labelled datasets are often scarce. Here, we developed a foundation model for cancer imaging biomarker discovery by training a convolutional encoder through self-supervised learning using a comprehensive dataset of 11,467 radiographic lesions. The foundation model was evaluated in distinct and clinically relevant applications of cancer imaging-based biomarkers. We found that it facilitated better and more efficient learning of imaging biomarkers and yielded task-specific models that significantly outperformed conventional supervised and other state-of-the-art pretrained implementations on downstream tasks, especially when training dataset sizes were very limited. Furthermore, the foundation model was more stable to input variations and showed strong associations with underlying biology. Our results demonstrate the tremendous potential of foundation models in discovering new imaging biomarkers that may extend to other clinical use cases and can accelerate the widespread translation of imaging biomarkers into clinical settings.

## Abstract (web summary)

Foundation models have transformed artificial intelligence by training on vast amounts of broad unlabelled data. Pai et al. present a foundation model leading to more accurate, efficient and robust cancer imaging biomarkers, especially in use cases with small training datasets.

## Main

Foundation models, popularized recently due to their unprecedented performance in language, vision and several other domains^1^, are large deep-learning models trained on extensive amounts of unannotated data serving as the base for a wide range of downstream tasks. In the field of natural language processing, for example, foundation models drive the successes of applications such as ChatGPT^2^, BERT^3^ and CLIP^4^. Similarly, foundation models, such as SimCLR^5^ and DINO^6^, have reported considerable success in computer vision applications.

Medicine represents a vast potential for foundation models as labelled data are scarce, while multimodal data, such as medical images, biologic and clinical notes, are frequently collected in routine clinical care^7^. Indeed, different applications of foundation models, such as augmented surgical procedures, bedside decision support, interactive radiology reports and note-taking, have been reported^8^.

While many studies investigating imaging-based biomarkers incorporate supervised deep-learning algorithms into their models^9–11^, they are typically applied in scenarios where large datasets are available for training and testing. The quantity and quality of annotated data are strongly linked to the robustness of deep-learning models. However, access to large amounts of annotated data for specialized applications is often challenging and demands expertise, time and labour. In such scenarios, many investigators fall back on traditional handcrafted or engineered approaches based on defined mathematical and statistical algorithms that analyse attributes such as the shape and texture of objects in images, which limit the scope of discovery. This caveat is commonplace in many scenarios where insights from imaging-based biomarkers have great potential in informing clinical care.

Foundation models are generally pretrained using self-supervised learning (SSL), a set of methods that leverage innate information available within data by learning generalized, task-agnostic representations from large amounts of unannotated samples. Existing literature^12^ has suggested several strategies, such as image reconstruction, to pretrain networks to learn these representations. Following pretraining, foundation models can be applied to task-specific problems, improving generalization, especially in tasks with small datasets. The expanding literature on SSL in medical imaging^13^ focuses primarily on two-dimensional (2D) images (X-ray, whole slide images, dermatology images, fundus images and so on) for diagnostic applications. There is still limited evidence investigating whether SSL can help train foundation models that learn general, robust and transferrable representations that can act as imaging biomarkers, especially prognostic, for tasks of clinical relevance.

**Fig. 1:**
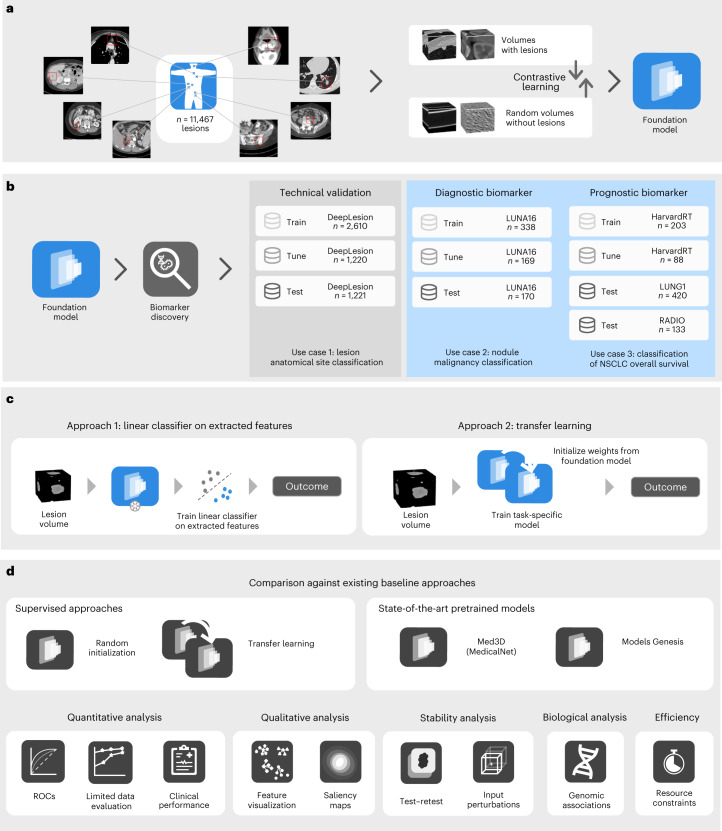
General overview of the study. **a**, Foundation model pretraining: a foundation model, specifically a deep convolutional encoder, was pretrained by contrasting volumes with and without lesions. **b**, Clinical application of the foundation model: the foundation model was used to extract biomarkers and subsequently evaluated on three classification tasks using diverse datasets. **c**, Foundation model implementation approaches: the foundation model was implemented on specific use cases by (1) training a linear classifier on extracted features or (2) through transfer learning by fine-tuning all model parameters. **d**, Performance evaluations: we compared the performance of the foundation model against supervised models, trained from random initialization and transfer-learned, through fine-tuning, from a different task. Publicly available state-of-the-art models, Med3D and Models Genesis, were also compared against our foundation model using identical implementation approaches. The comparison was made through several criteria for the different use cases, including quantitative performance, stability, biological and efficiency analysis.

In this study, we investigated whether foundation models can improve the development of deep-learning-based imaging biomarkers, especially in limited dataset-size scenarios. The foundation model, a convolutional encoder, was self-supervised pretrained on 11,467 diverse and annotated lesions identified on computed tomography (CT) imaging from 2,312 unique patients^14^ (Fig. 1a). The model was first technically validated by classifying lesion anatomical site (use case 1). Subsequently, it was applied to two clinically relevant applications: developing a diagnostic biomarker that predicts the malignancy of lung nodules (use case 2) and a prognostic biomarker for non-small cell lung cancer (NSCLC) tumours (use case 3; Fig. 1b). We evaluated two distinct implementation approaches of incorporating a pretrained foundation model into training pipelines for downstream tasks: using the foundation model as a feature extractor followed by a linear classifier and another where the foundation model is fine-tuned through transfer learning. The performance of the foundation model approaches was compared to several existing baselines developed using supervised approaches and publicly available pretrained models. Our analysis examines effective pretraining techniques, performance in limited data scenarios, consistency in test–retest and inter-reader evaluations and the interpretability of findings through deep-learning attribution methods along with their biological relevance to gene expression data. Our results demonstrate the potential of foundation models in discovering new imaging biomarkers and their particular strength in applications with limited dataset sizes. This evidence may extend to other clinical use cases and imaging modalities and can accelerate the widespread development and translation of imaging biomarkers into clinical settings.

## Results

We developed a deep-learning foundation model using SSL and tested the model’s performance in three distinct use cases. The study design and the pretraining process are outlined in Fig. 1. We trained a single foundation model using a dataset with 11,467 annotated CT lesions identified from 2,312 unique patients. Lesion findings were diverse and included multiple lesions, such as lung nodules, cysts and breast lesions, among numerous others. A task-agnostic contrastive learning strategy was used to pretrain the model on these lesion findings (Fig. 1a). We showed the applicability of our pretrained foundation model to several tasks through the evaluation on three diverse clinical applications over five distinct datasets (Fig. 1b).

### Pretraining strategy selection

**Fig. 2:**
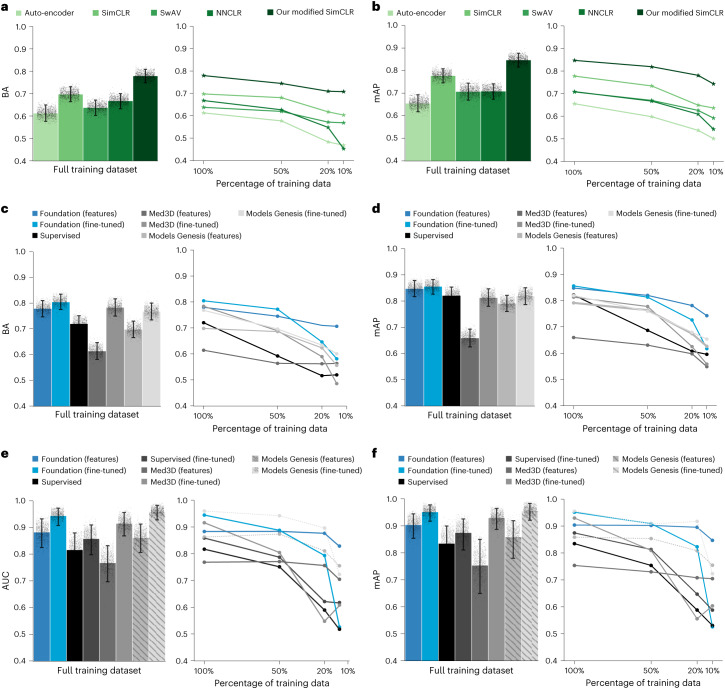
Comparison of pretraining strategies and performance evaluation for lesion anatomical site (use case 1) and nodule malignancy classification (use case 2). We determined the best pretraining approach for our foundation model on their ability to extract features that can be linearly classified to best predict lesion anatomical site. **a**,**b**, Different pretraining approaches were evaluated using balanced accuracy (BA) (**a**) and mAP (**b**). **c**,**d**, After pretraining our foundation model using the best strategy, we adapted them to use case 1, lesion anatomical site classification, and compared them against baseline methods using balanced accuracy (**c**) and mAP (**d**). We show performance on these metrics aggregated across eight anatomical sites when trained on the full training set and when the training data percentage decreased to 50, 20 and 10%. **e**,**f**, Similar to use case 1, we implemented our foundation model on use case 2 and compared it against baseline methods using the AUC-ROC (**e**) and mAP (**f**). Both metrics were computed when trained on the full and 50, 20 and 10% of the dataset. In **e**,**f**, Models Genesis approaches are shaded and/or dotted as they were trained on the same data split of LUNA16 and therefore do not present a fair comparison due to overfitting. For use case 2, we also added a supervised model fine-tuned through transfer learning from use case 1. The error bars for **a**–**f** show 95% CIs of the estimates and the bar centre shows the mean estimate of the displayed metric. The estimates were computed by generating a bootstrap distribution with 1,000 resamples for datasets with *n* = 1,221 samples (**a**–**d**) and *n* = 170 samples (**e**,**f**).

We compared simple auto-encoder pretraining and several state-of-the-art self-supervised pretraining approaches—namely SimCLR^5^, SwAV^15^ and NNCLR^16^—against the modified version of SimCLR developed in our study (Methods). We evaluated pretraining strategies on the technical validation use case of lesion anatomical site classification by comparing linear classifiers trained on top of features extracted from each of the chosen strategies. We observed that our modified SimCLR pretraining surpassed all others (*P* < 0.001) in balanced accuracy (Fig. 2a) and mean average precision (mAP) (Fig. 2b), achieving a balanced accuracy of 0.779 (95% confidence interval (CI) 0.750–0.810) and mAP = 0.847 (95% CI 0.750–0.810). As expected, the second best-performing approach was SimCLR (balanced accuracy 0.696 (95% CI 0.663–0.728); mAP = 0.779 (95% CI 0.749–0.811)). The auto-encoder approach, previously popular for pretraining, performed the worst compared to state-of-the-art contrastive SSL approaches.

When limited data (50, 20 and 10%) was used for downstream task training, our method demonstrated consistently improved performance. More importantly, it remained robust as evidenced by the smallest decline in balanced accuracy and mAP of 9 and 12%, respectively, when reducing training data from 100 to 10%.

### Lesion anatomical site classification (use case 1)

As a technical validation of the foundation model, we selected an in-distribution task (that is, sourced from the same cohort as the foundation model pretraining) and developed classification models to predict anatomical sites on a training and tuning dataset totalling 3,830 lesions (use case 1, Fig. 1b). On a held-out test set of 1,221 lesions, we evaluated the performance of two different implementations of the foundation model (Fig. 1c).

We found that foundation model implementations showed superiority over compared baseline methods (Fig. 2c,d). The fine-tuned foundation model, denoted Foundation (fine-tuned), with a mAP of 0.857 (95% CI 0.828–0.886) significantly (*P* < 0.05) outperformed all baseline methods on mAP. With a balanced accuracy of 0.804 (95% CI 0.775–0.835), a significant (*P* < 0.01) improvement in balanced accuracy was also observed in comparison to all baselines except Med3D (fine-tuned), where the improvement was borderline (*P* = 0.059).

**Extended Data Fig. 1:**
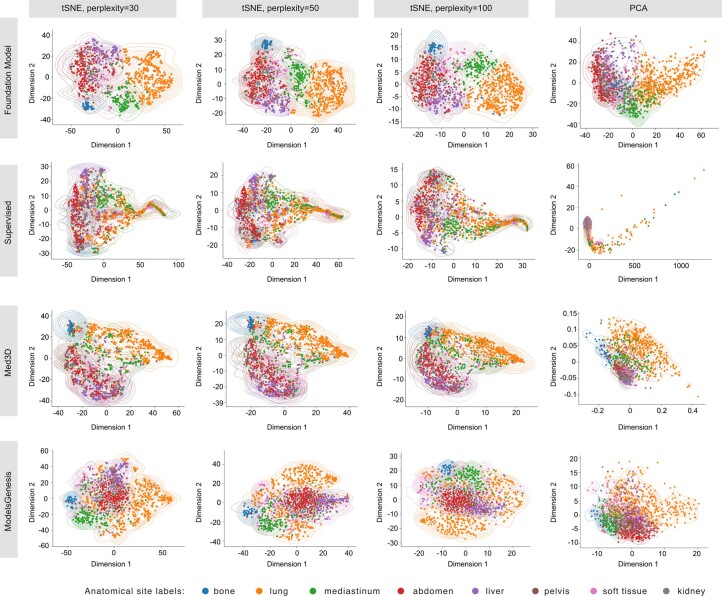
Visual exploration of the features generated from the foundation and baseline models. Features from the foundation model and each of the baseline models are extracted on the independent test-set for identifying lesion anatomical sites and visualized using several different dimensionality reduction approaches. Approaches chosen aim to avoid biases from parameter selection, therefore, tSNE with different perplexity settings and PCA are used. The x-axis corresponds to dimension 1, and the y-axis to dimension 2 of the dimensionality reduction. The density contours of each class are underlaid to highlight separability between classes in the feature space. It is to be noted that the supervised model was trained with lesion anatomical site labels while all the other models (Foundation, Med3D, ModelsGenesis) were used merely as feature extractors without being trained for the particular label.

**Extended Data Fig. 2:**
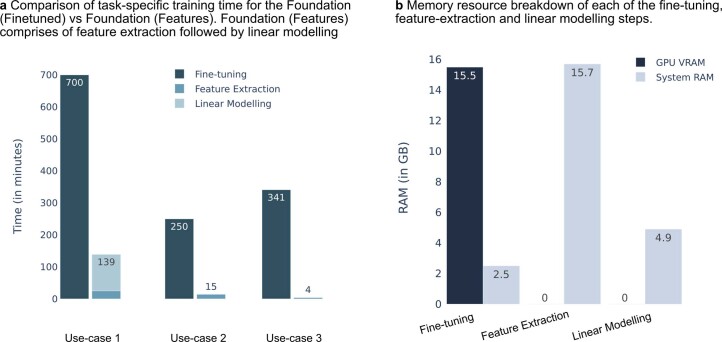
Time and memory efficiency of implementation approaches. We compare the two implementation approaches of our foundation model, 1) linear modelling on extracted features, which comprises a feature extraction step followed by the linear modelling step, and 2) transfer learning through a fine-tuning step. **a**, Training times (in minutes) for each of the three use cases and the three steps are shown. **b**, Memory usage (GPU VRAM and System RAM) are shown for the feature extraction, linear modelling and fine-tuning steps. Memory usage for each step across use-cases remains mostly constant due to batch processing and high feature dimensionality. All analyses were run with six cores on AMD EPYCTM 7402 P Processor 24-core @ 2.80 GHz. The GPU, which was only used for fine-tuning, was the Quadro RTX 8000. For both CPU and GPU runs, where batch processing was used, a batch size of 32 was chosen.

Features extracted from the foundation model, Foundation (features), when linearly classified, showed significantly improved performance in balanced accuracy and mAP over features extracted from Med3D (ref. ^17^) and Models Genesis^18^ baseline methods. Models fine-tuned using compute-intensive supervised deep-learning methods—Supervised, Med3D (fine-tuned) and Models Genesis (fine-tuned)—did not significantly improve in balanced accuracy and mAP over the simple linear classification of foundation model features. Moreover, when considering only mAP, the simple linear classification significantly (*P* < 0.05) outperformed all other implementations. To provide deeper insight into feature separability that allows for such strong linear classification performance, we attempted to explore visual associations by interpreting projected features (Extended Data Fig. 1). We observed that features from the pretrained foundation model provided consistently interpretable and well-separated clusters across different settings. Modelling using features also provided a computational benefit, with both memory and time, over deep-learning training (Extended Data Fig. 2).

**Extended Data Fig. 3:**
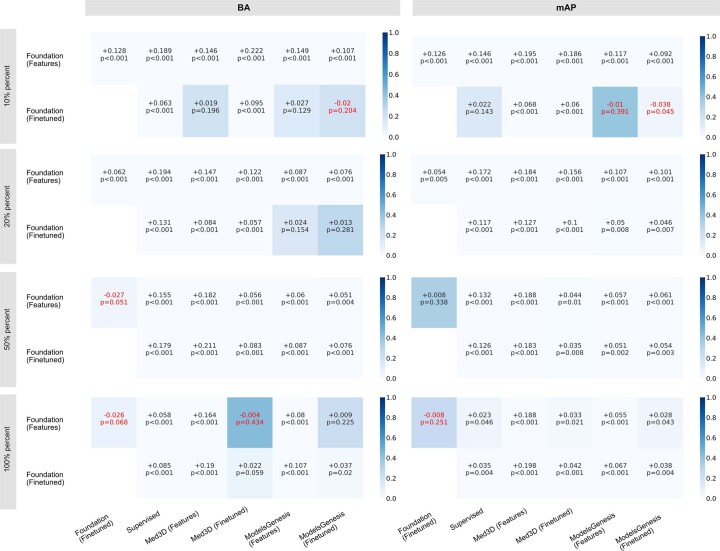
Detailed comparison of the foundation model implementations against baseline methods for lesion anatomical site classification. Comparison of the balanced accuracy and mean average precision of the Foundation (Features) and Foundation (Finetuned) against all other methods when using 100%, 50%, 20%, and 10% percent of the training data. For each metric-percentage pair, a p-value heatmap (darker colours show non-significant values) is shown with the foundation models on the y-axis and all other models to compare on the x-axis. In each cell, the increase or decrease in metric value is shown along with the corresponding p-value. p-values between models were compared using the permutation test with N = 1000 permutations conducted for each pair-wise comparison.

**Extended Data Fig. 4:**
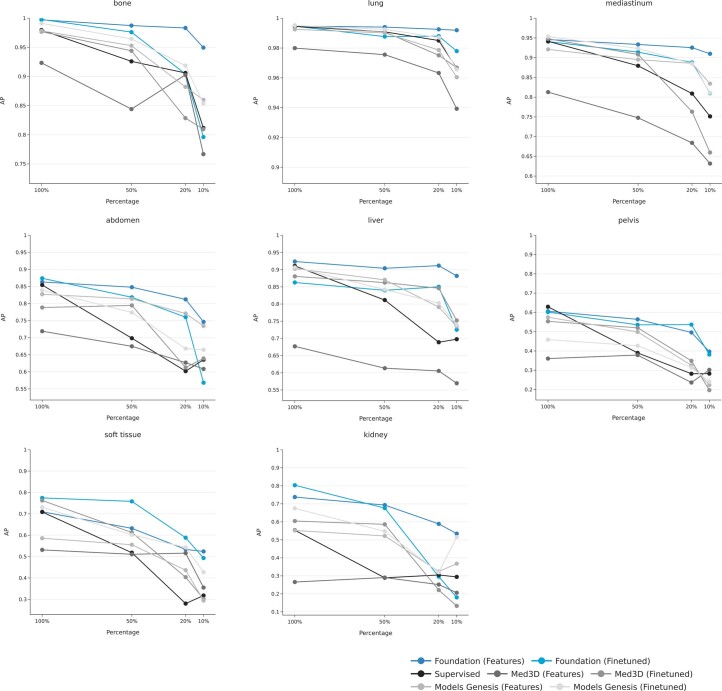
Anatomical site-wise breakdown of foundation model and baseline method performance. We compare the foundation model against baseline methods across different training data percentages using average precision scores for each anatomical site in the DeepLesion held-out test dataset. This allows us to show the generalizability of approaches across anatomical sites.

The performance advantage of the foundation model was even stronger in limited data scenarios (Fig. 2c,d). When we reduced training data to 50% (*n* = 2,526), 20% (*n* = 1,010) and 10% (*n* = 505), Foundation (features) significantly improved balanced accuracy and mAP over every baseline method. Foundation (fine-tuned) showed a larger drop in performance and failed to improve significantly over baseline implementations as training data were decreased (losing significance from 20% onward). Individual comparisons between each model can be found in Extended Data Fig. 3. To show the applicability of our approach across the various anatomical sites, we provide a site-wise breakdown of performance in Extended Data Fig. 4.

### Nodule malignancy prediction (use case 2)

To assess the generalizability of the foundation model, we chose an out-of-distribution task (that is, belonging to a cohort different from the pretraining) and trained classification models to predict the malignancy of 507 lung nodules from the LUNA16 dataset (use case 2 in Fig. 1b). We then evaluated performance on a separate test set of 170 nodules.

The approach of fine-tuning the foundation model, Foundation (fine-tuned), with an area under the curve (AUC) = 0.944 (95% CI 0.907–0.972) and mAP = 0.953 (95% CI 0.915–0.979) resulted in significant (*P* < 0.01) superiority over most of the baseline implementations (Fig. 2e,f). The implementation Med3D (fine-tuned), with AUC = 0.917 (95% CI 0.871–0.957) and mAP = 0.9307 (95% CI 0.888–0.964), performs slightly worse than our model, but this is not significant (*P* = 0.134). For features extracted from our foundation model, similar to use case 1, our implementation surpasses (*P* < 0.001) baseline feature-based implementations. Notably, none of the deep-learning fine-tuned baselines significantly improve over linear classification. The baseline Models Genesis implementation was excluded in this analysis as this model was pretrained on the same dataset and, therefore, does not indicate a fair comparison.

**Extended Data Fig. 5:**
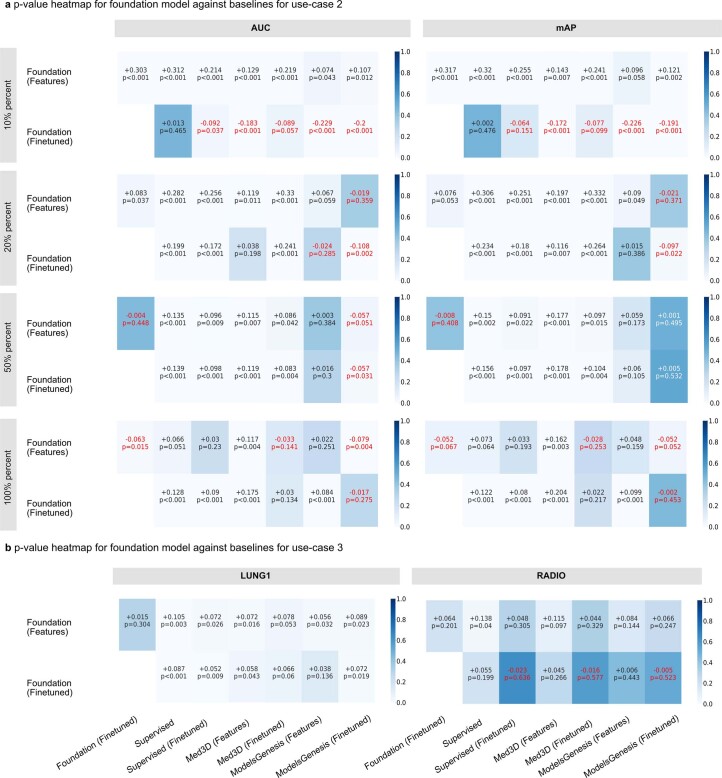
Detailed comparison of the foundation model implementations against baseline methods for nodule malignancy classification and NSCLC prognostication. **a**, Comparison of the area-under-receiver operating curve (AUC) and mean average precision(mAP) of the Foundation (Features) and Foundation (Finetuned) against all other methods when using 100%, 50%, 20%, and 10% percent of the training data on use case 2. **b**, Comparison of the AUC of the Foundation (Features) and Foundation (Finetuned) against all other models for the LUNG1 (left) and RADIO (right) dataset for use-case 3. For each metric-percentage pair, a p-value heatmap (darker colours show non-significant values) is shown with the foundation models on the y-axis and all other models to compare on the x-axis. In each cell, the increase or decrease in metric value is shown along with the corresponding p-value. p-values between models were compared using the permutation test with N = 1000 permutations conducted for each pair-wise comparison.

Again, the Foundation (features) approach shows improved performance in reduced data analyses, dominating all baselines (*P* < 0.05) on 50% (*n* = 254), 20% (*n* = 101) and 10% (*n* = 51) training data. Foundation (fine-tuned) shows superior performance over all baselines at 50% but shows large drops in performance from a 20% reduction onward. Med3D (fine-tuned), which performed well on the full dataset, shows a large drop from 50% data reduction onward. Detailed comparisons can be found in Extended Data Fig. 5a.

### NSCLC prognostication (use case 3)

Next, we evaluated the efficacy of our foundation model in another clinically relevant use case to capture prognostic radiographic phenotypes of NSCLC tumours. We trained and tuned prognostication models using data from the HarvardRT (*n* = 291) cohort to predict 2 year overall survival after treatment and then compared the performance of the foundation model and baseline implementations on two independent testing cohorts, LUNG1 (NSCLC-Radiomics) (*n* = 420) and RADIO (NSCLC-Radiogenomics) (*n* = 133) (use case 3 in Fig. 1b).

**Fig. 3:**
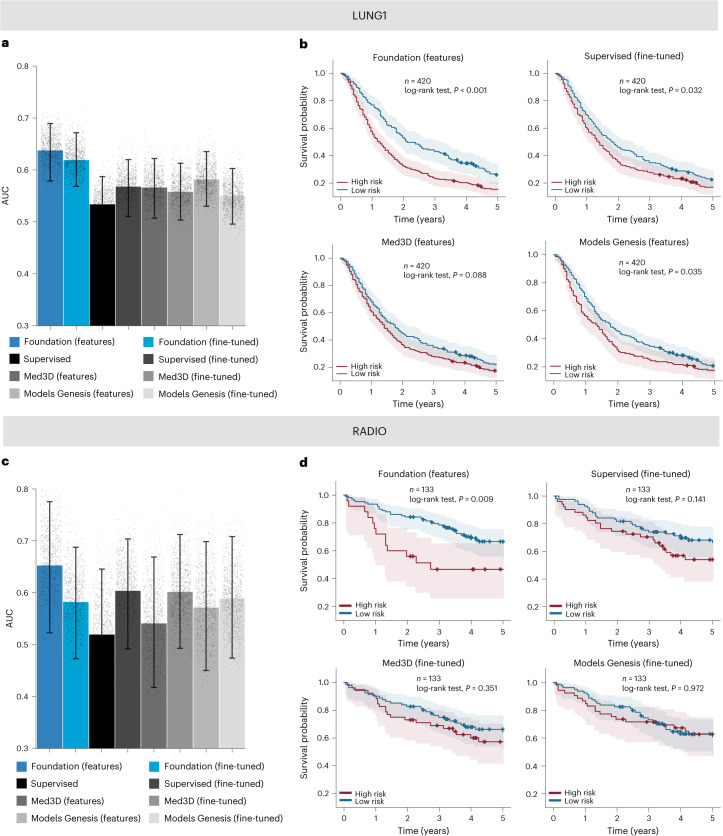
Performance of the foundation model for prognostication of NSCLC tumours (use case 3). We compared the foundation model implementation approaches against baseline methods using the AUC. **a**,**c**, Each implementation was adapted for 2 year overall survival classification, trained on the HarvardRT dataset and evaluated on LUNG1 (**a**) and RADIO (**c**) datasets. **b**,**d**, Kaplan–Meier curves for groups stratified by model predictions from the best performing among implementation approaches are shown for LUNG1 (**b**) and RADIO (**d**). To ensure a fair comparison, we calculated the threshold to split the risk groups on the HarvardRT tuning set for each implementation. Kaplan–Meier curves for all approaches can be found in Extended Data Fig. 6. The 95% CI of the estimates is shown by error bars in **a**,**c** and error bands in **b**,**d**. The measure of centre for the error bars is the mean estimate of AUC and the measure of centre for the error bands is the Kaplan–Meier estimate of the survival function. The estimates for the bar plots in **a** and **c** have been computed through a bootstrap distribution with 1,000 resamples using dataset sizes of *n* = 420 and *n* = 133, respectively.

**Extended Data Fig. 6:**
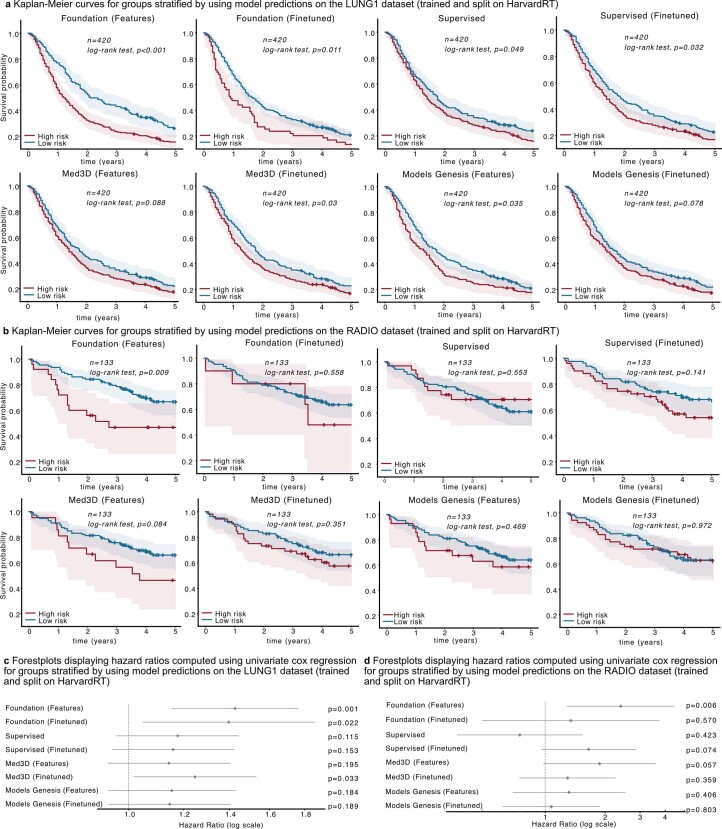
Survival analysis for all models implemented on NSCLC prognostication. **a**,**b**, Kaplan Meier curves on the LUNG1 (**a**) and RADIO (**b**) datasets for both the foundation model implementation approaches as well as the baseline comparisons are shown. **c**,**d**, In **c** and **d**, Hazard ratios (HR), computed through univariate Cox regression, for each of the implementation approaches on the LUNG1 and RADIO datasets are shown using forest plots. For both these analyses, groups are determined based on respective model predictions split on the median of the corresponding HarvardRT tuning set predictions. The error bands in (a,b) represent the 95% confidence interval of the Kaplan-Meier estimates of the survival function. The log-rank test is used to determine significant differences between the groups in the KM analysis. For (**c**, **d**), the error bars represent the 95% confidence interval of the hazard ratio, and the p-values are calculated using the Wald test. For the LUNG1 dataset, n = 420 and RADIO, n = 133 samples are used to compute each of the analyses in the plots above.

In the LUNG1 cohort, features extracted from the foundation model followed by a linear classifier, Foundation (features), exceeded all baseline performances with an AUC of 0.638 (95% CI 0.584–0.692) (Fig. 3a). All comparisons were significant (*P* < 0.05) except for Med3D (fine-tuned), where borderline significance was observed (*P* = 0.053). Deep-learning-based implementations in the baseline comparisons did not perform strongly on this use case. In addition to AUC, we plotted Kaplan–Meier estimates for the top-performing implementations (Fig. 3b). Foundation (features) provided the best stratification (*P* < 0.001), indicating its ability to determine appropriate risk groups on the basis of mortality. More detailed analyses can be found in Extended Data Figs. 5b and 6.

For the RADIO cohort, Foundation (features) shows the best performance with an AUC of 0.653 (95% CI 0.532–0.771). Similar to the LUNG1 cohort, deep-learning implementations did not demonstrate superior performance (Fig. 3c). Due to the small sample size, none of the models showed significant differences from the rest (*P* > 0.05) except for the Foundation (features) improving over the Supervised model, which had near-random performance (AUC = 0.520). Kaplan–Meier analysis showed that the sole model that offered significant stratification was the Foundation (features) with *P* = 0.009 (Fig. 3d).

### Stability of the foundation model

**Fig. 4:**
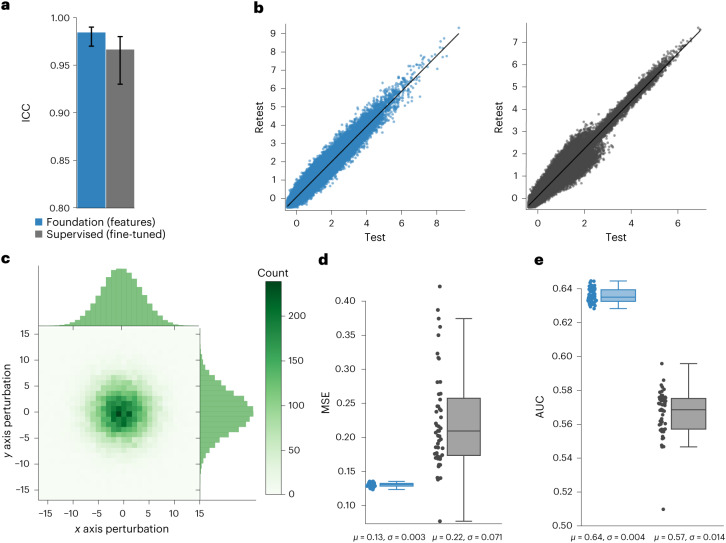
Input and test–retest stability of the foundation model. We analysed input stability on the LUNG1 dataset and test–retest robustness on the RIDER dataset by comparing between Foundation (features) and Supervised (fine-tuned) (best performing, overall for LUNG1 and RADIO use cases). **a**, We compared ICC between test–retest model predictions on the RIDER dataset (*n* = 26). **b**, We further visualize the linearity between flattened features extracted from test and retest scans on the RIDER dataset. **c**, We show the sampling distribution for input perturbations that are used to simulate inter-reader variability. We perturbed across *x*, *y* and *z* axes, although the distribution is shown only for *x* and *y* perturbations for simplicity. **d**, We compared the stability of the features across models using mean-squared error (MSE) between feature values across all the trials. **e**, We demonstrated the prognostic stability of models when the input seed point is perturbed, estimated through calculating AUC for 2-year survival from model predictions. The error bars in **a** represent the 95% CI of the estimates and the bar centre is the mean estimate. For the box plots (**d**,**e**), the centre line shows the median, the box edges represent first and third quartiles and the whiskers extend to 1.5 times the inter-quartile range. The distribution of the data is shown alongside the box plot. Each AUC and MSE measure in the box plots (**d**,**e**) have been computed on a dataset with *n* = 422 samples and the distribution of the measures are obtained from 50 independent perturbation trials.

We evaluated the stability of our foundation model through a test–retest scenario and an inter-reader variability analysis. We used scans from 26 patients from the RIDER dataset^19^, routinely used for test–retest robustness analysis in tumour imaging^19–21^. We found that predictions from the overall best-performing models on LUNG1 and RADIO: Foundation (features) and Supervised (fine-tuned) had high stability with intraclass correlation coefficient (ICC) values of 0.984 and 0.966, respectively. Furthermore, the test–retest features for both networks were strongly correlated (Fig. 4a,b).

To evaluate stability against inter-reader variability, we used the LUNG1 dataset and perturbed the input seed point to extract the three-dimensional (3D) volume, simulating variations among human readers (Fig. 4c). We found that the Foundation (features) had significantly (*P* < 0.05) higher stability against simulated inter-reader variations in feature differences and prediction performance (Fig. 4d,e).

### Saliency maps for fine-tuned foundation models

**Fig. 5:**
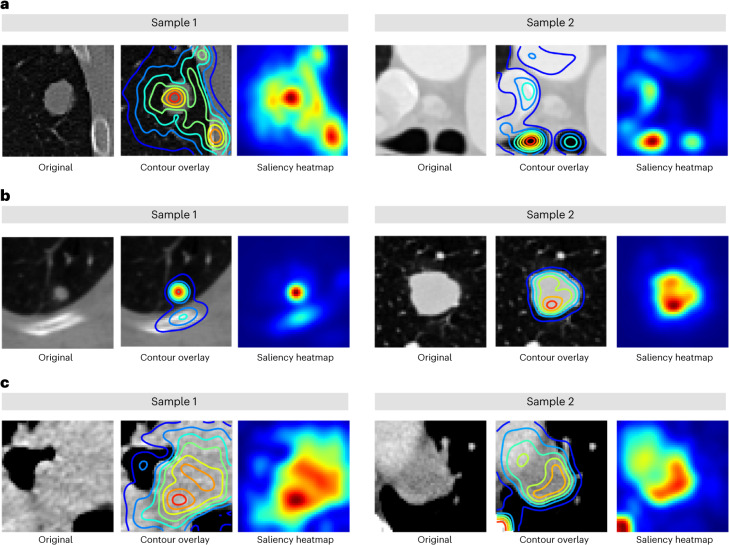
Saliency maps for fine-tuned foundation models. **a**–**c**, We generated gradient-based saliency maps for each of the fine-tuned foundation models from use cases 1 (**a**), 2 (**b**) and 3 (**c**) using smooth guided back-propagation and visualized salient regions on two samples from corresponding test datasets. The first and fourth columns show the central axial slice (50 × 50 mm) of the volume provided as input to the model. The second and fifth columns show isolines for saliency contours overlayed on the image. Finally, the third and sixth columns show saliency maps highlighting areas of the input volume that contribute the most to a change in the output prediction.

To gain insight into regions of the input volumes that contribute to a given prediction, we used gradient-based saliency maps for Foundation (fine-tuned) on three selected use cases (as depicted in Fig. 5).

Our analysis revealed that for each use case, the focus was primarily around tissues within or in proximity to the tumour, which is consistent with research demonstrating the tumour microenvironment’s influence on cancer development^22^ and prognosis. Specifically, in use case 1 (Fig. 5a), the focus was mainly on areas surrounding the lesions, such as the parenchyma and bone regions in the lung and the trachea in mediastinal lesions. For use case 2 (Fig. 5b), tissues of the nodule were highlighted, avoiding high-density bone regions. Use case 3 (Fig. 5c) primarily attributed areas surrounding the centre of mass of the tumour, with some contribution from high-density bone regions. Overall, these findings indicated that the areas that contribute to the networks’ predictions varied in accordance with the specific use case, with the tumour and surrounding tissues playing a pivotal role.

### Underlying biological basis of the foundation model

**Fig. 6:**
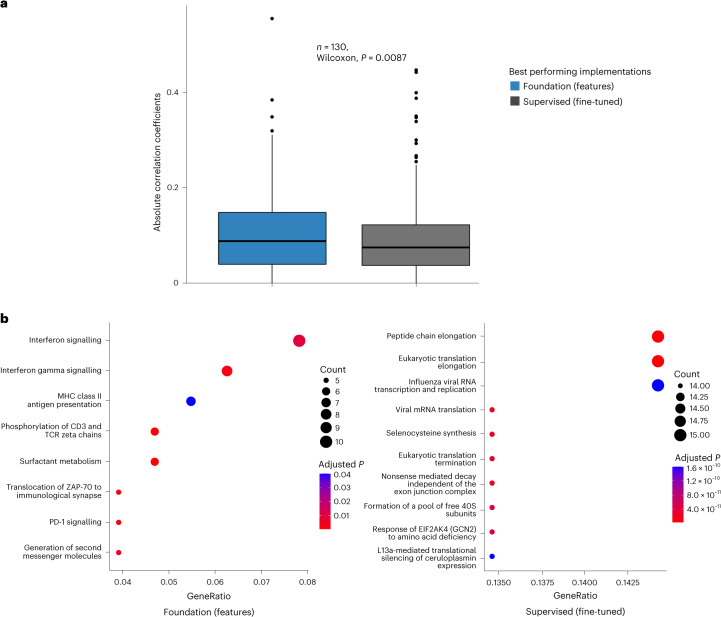
Underlying biological basis of the foundation model. We compared the Foundation (features) and Supervised (fine-tuned) (best-performing models on the RADIO dataset) model predictions with gene expression profiles. **a**, Box plot of absolute correlation coefficients (*y* axis) of selected genes against model predictions (*x* axis) across *n* = 130 samples. Statistical significance between the two groups is determined through a two-sided Wilcoxon signed rank test. **b**, Gene-set enrichment analysis of genes with correlation coefficient greater than 0.1 revealed for the foundation (left) and supervised model predictions (right). Genetic pathways are shown on the *y* axis, and the gene ratio is shown on the *x* axis. Gene count and adjusted *P* values are also shown in the legend. False discovery rates are used to adjust the *P* values for multiple comparisons. The box plots in **a** are defined by the median as the centre line, first and third quartiles as the box edges and 1.5 times the inter-quartile range as the whiskers. MHC, major histocompatibility complex.

Finally, we investigated the biological basis of our foundation model by analysing gene expression data associated with model predictions for 130 participants from the RADIO dataset. To identify relevant genes, we selected the top 500 genes and performed a correlation analysis, comparing Foundation (features) and Supervised (fine-tuned) predictions with gene expression profiles. We found that absolute correlation coefficients between gene expression profiles and model predictions were significantly higher (*P* = 0.008) for the foundation model, indicating a stronger association with underlying tumour biology (Fig. 6a).

Additionally, we examined the genes associated with these models through a gene-set enrichment analysis (genes with a correlation coefficient >0.1). Our analysis revealed that the foundation model showed an enrichment pattern of immune-associated pathways, including interferon signalling, interferon gamma signalling, major histocompatibility complex class II antigen presentation and PD-1 signalling. Conversely, while the supervised model did show enrichment of individual pathways, no identifiable pattern was observed (Fig. 6b).

## Discussion

In this study, we demonstrated that our foundation model, trained using self-supervised contrastive learning, provided robust performance in predicting anatomical site, malignancy and prognosis across three different use cases in four cohorts. Several studies^23–25^ have demonstrated the efficacy of SSL in medicine where only limited data might be available for training deep-learning networks. Our findings complement and extend this for identifying reliable imaging biomarkers for cancer-associated use cases. We showed that our foundation model provided superior performance for anatomical lesion site classification on average and across individual anatomical sites, even when very few training samples were available for that site. Similarly, for malignancy prediction, our model outperformed all other baseline approaches. In both these use cases, the benefit of our model was especially evident in limited data scenarios. Modelling using features extracted from the foundation model was the most robust across these use cases when subjected to drops in training data, offering stable performance even when data sizes were considerably reduced, for example, using only 51 samples in use case 2. Using these features provided the best performance on small cohorts in predicting prognosis and also demonstrated significant stratification of patients by their associated risk for each of the LUNG1 and RADIO cohorts (*P* < 0.01). Feature-based implementations were also computationally efficient when considering both time and memory. Additionally, features and predictions from the foundation model features were found to be highly stable against inter-reader and test–retest variations. Regarding interpretability, we observed that models focused on varying regions of the tumour and surrounding tissue relevant to the associated use case. To gain insight into the underlying biological associations of these features, RNA sequencing analysis combined with imaging data showed that these features correlated with immune-associated pathways.

Image-biomarker studies for predicting endpoints, such as overall survival on small cohorts, largely rely on statistical feature extraction (engineered radiomics) and classical machine learning-based modelling. These require precise 3D segmentations for feature extraction, increasing the annotation burden of these studies. Moreover, these statistical features are affected by several confounders, such as inter-reader variability in segmentations^26^ and acquisition settings of the scanners^27^, limiting their applicability in diverse settings. Deep-learning methods, in comparison, are robust to differences in acquisition and segmentation variability and provide improved performance^10^. Surveying diagnostic biomarker studies, Shen et al.^28^ trained a simple deep convolutional network to extract features from lung nodules followed by malignancy classification using a support vector machine, possibly one of the first convolutional approaches for this use case. In a subsequent study, Shen et al.^29^ proposed a new multi-crop convolutional neural networks (CNN) architecture and demonstrated improved performance over auto-encoder-based pretraining and radiomic feature-based training. Kumar et al.^30^ identified radiomic sequences through deep convolutional encoders to determine lung nodule malignancy. These developed approaches were specific to nodule malignancy classification, and it is difficult to determine their transferability to other use cases. By contrast, our approach is generalizable to multiple use cases, and for nodule malignancy, we obtain high performance using significantly lesser training data, only 338 nodules (due to our more stringent exclusion criteria). Considering prognostic biomarkers, Hosny et al.^10^ trained a deep-learning model for lung cancer prognostication using several multi-institutional cohorts and demonstrated strong performance over traditional radiomics. Haarburger et al.^31^ presented a deep convolutional network-based approach to predict survival endpoints on the LUNG1 dataset. Mukherjee et al.^32^ developed a shallow CNN for predicting overall survival by round-robin training on four different cohorts and additionally observed that their model transferred well to predicting nodule malignancy. A general trend observed across these studies was that the performance of deep-learning models was more robust when larger and multi-institutional cohorts were available for training, and validation was generally performed on smaller cohorts. A demonstrated strength of our approach is that training on smaller cohorts performs well in larger validation cohorts.

Advances in deep learning, such as SSL, have translated well to medical imaging use cases, with several studies incorporating pretraining for improved performance^23,25,33,34^. More recently, foundation models have become popular for their ability to learn general concepts adaptable to various tasks. Zhou et al.^35^ proposed a foundation model where a visual transformer was trained on 1.6 million retinal images and validated on ocular disease use cases. Azizi et al.^36^ presented distinct foundation models for five domains trained in a multi-step approach with different amounts of pretraining data for each (ranging from 8,000 to 2.2 million images). Azad et al.^37^ conducted an extensive review, highlighting the development of diverse foundation models, both generalist and more specific, across several medical imaging domains.

Developing a reliable and reproducible foundation model for a specific domain involves the consideration of several design choices. Cole et al.^38^ present empirical observations on the quantity of pretraining data, the impact of the pretraining domain, the quality of data and task difficulty when using contrastive pretraining methods. They show a saturation point associated with pretraining dataset size and diminishing returns beyond this point. This point largely depends on the nature and sizes of training data in the downstream task. In our study, we pretrained on 11,467 lesion volumes and randomly sampled volumes, from 5,513 unique CT scans, leveraging not only one of the largest lesion-specific datasets but also one of the largest pretraining 3D CT datasets. The only other study we know that uses more data is by Ghesu et al.^25^ where 24,000 CT scans are used for pretraining. Cole et al.^38^ also showed that pretraining using in-domain data, semantically connected to the downstream task, has a huge impact besides scale of the pretraining data. Azizi et al.^36^ also observed improvements when incorporating in-domain data, even when the number of samples used was smaller. In the context of our study, our pretraining process is the closest to the domain of oncological image biomarkers; as a result, improvements over more out-of-domain pretraining methods are seen.

Despite the strengths outlined in our study, we recognize several limitations that need to be addressed before the clinical applicability of our foundation model. First, the retrospective nature of this study constrains our ability to assess the real-world practicality of model-based biomarkers. Second, evaluating the model’s reliability and reproducibility across diverse demographic groups and various biomarker discovery tasks is crucial to ensure broad applicability. This includes examining how well the model handles distribution shifts between the pretraining and application phases. Another key consideration is investigating whether a larger volume of pretraining data could enhance model performance, particularly for complex tasks. Additionally, since imaging features alone may not suffice for comprehensive clinical decision making, integrating clinical data as covariates could notably improve the model’s effectiveness. Third, a significant challenge with deep-learning models, including ours, is their ‘black box’ nature, which limits interpretability and explainability. Although we used established saliency attribution methods to interpret our model’s predictions, the technical limitations^39,40^ of these methods may restrict the applicability of the insights gained. Furthermore, our initial biological association analysis, aimed at explaining the model’s decisions, is preliminary and requires more rigorous investigation for a concrete understanding.

In conclusion, our foundation model offers a powerful and reliable framework for discovering cancer imaging biomarkers, especially in small datasets. Furthermore, it surpasses current deep-learning techniques in various tasks while fitting conveniently into existing radiomic research methods. This approach can potentially uncover new biomarkers contributing to research and medical practice. We share our foundation model and reproducible workflows so that more studies can investigate our methods, determine their generalizability and incorporate them into their research studies.

## Methods

### Study population

We use a total of five distinct datasets: four of which are publicly accessible and one is an internal dataset. These were acquired from various institutions as components of separate investigations (Extended Data Fig. 9).

DeepLesion^14^ is a dataset comprising 32,735 lesions from 10,594 studies of 4,427 unique patients collected over two decades from the National Institute of Health Clinical Center PACS server. Various lesions, including kidney, bone and liver lesions, as well as enlarged lymph nodes and lung nodules, are annotated. The lesions are identified through radiologist bookmarked RECIST (Response Evaluation Criteria in Solid Tumors, National Cancer Institute, USA) diameters across 32,120 CT slices. In our study, we excluded CT scans with a slice thickness exceeding 3 mm, resulting in 16,518 remaining lesions. Subsequently, we divided this into 11,467 unlabelled lesions for contrastive training and 5,051 labelled lesions for anatomical site classification. The unlabelled lesions were sourced from 5,513 unique CT scans across 2,312 patients. Labelled lesions chosen for the anatomical site classification use cases were excluded from the pretraining data to avoid potential data leakage between pretraining and evaluation tasks. Despite not using class labels during pretraining, we consciously decided to prevent overlapping lesions from being seen at this stage to ensure unbiased evaluation. The labelled lesion data were further separated randomly into training, tuning and testing sets, containing 2,610, 1,220 and 1,221 lesions, respectively.

LUNA16 (ref. ^41^) is a curated version of the LIDC-IDRI dataset of 888 diagnostic and lung cancer screening thoracic CT scans obtained from seven academic centres and eight medical imaging companies comprising 1,186 nodules. The nodules are accompanied by annotations agreed on by at least three out of four radiologists. Alongside nodule location annotations, radiologists also noted various observed attributes such as internal composition, calcification, malignancy, suspiciousness and more. For our evaluation, we chose nodules with at least one indication of malignancy suspicion, totalling 677. We randomly picked 338 nodules for training and 169 for tuning the malignancy prediction networks. The final 170 nodules were used to assess the networks’ performance.

HarvardRT^10^ is a cohort of 317 patients with stage I–IIIB NSCLC treated with radiation therapy at the Dana-Farber Cancer Institute and Brigham and Women’s Hospital, Boston, MA, USA, between 2001 and 2015. All CT scans for this cohort were acquired with and without intravenous contrast on the GE Lightspeed CT scanner. The primary tumour site was contoured by radiation oncologists using soft tissue and lung windows. A subset of 291 patients with a follow-up of 2 years was selected for this study. We used 203 tumour volumes for training the prognostication networks and the remaining 88 tumour volumes for tuning.

LUNG1 (ref. ^42^) is a cohort of 422 patients with stage I–IIIB NSCLC treated with radiation therapy at MAASTRO Clinic, Maastricht, the Netherlands. Fluorodeoxyglucose positron emission tomography (PET)-CT scans were acquired with or without contrast on the Siemens Biograph Scanner. Radiation oncologists used PET and CT images to delineate the gross tumour volume. For our study, we selected CT scans of 420 patients (right-censored for 2-year survival) with annotated primary gross tumour volumes and used these as an independent test set for prognostication networks.

The RADIO^43^ dataset is a collection of 211 patients with NSCLC stage I–IV recruited between 2008 and 2012 who were referred for surgical treatment and underwent preoperative CT and PET-CT scans. These patients were recruited from the Stanford University School of Medicine and the Palo Alto Veterans Affairs Healthcare System. Scans were obtained using various scanners and protocols depending on the institution and physician. A subset of 144 patients in the cohort have available tumour segmentations independently reviewed by two thoracic radiologists. In addition to imaging data, the dataset includes molecular data from EGFR, KRAS, ALK mutational testing, gene expression microarrays and RNA sequencing. For the current study, we used 133 patients with annotated gross tumour volumes as an independent test set for prognostication after right-censoring for 2 year survival and subsequently investigated the biological basis of our networks using this dataset.

### Data preprocessing

**Extended Data Fig. 7:**
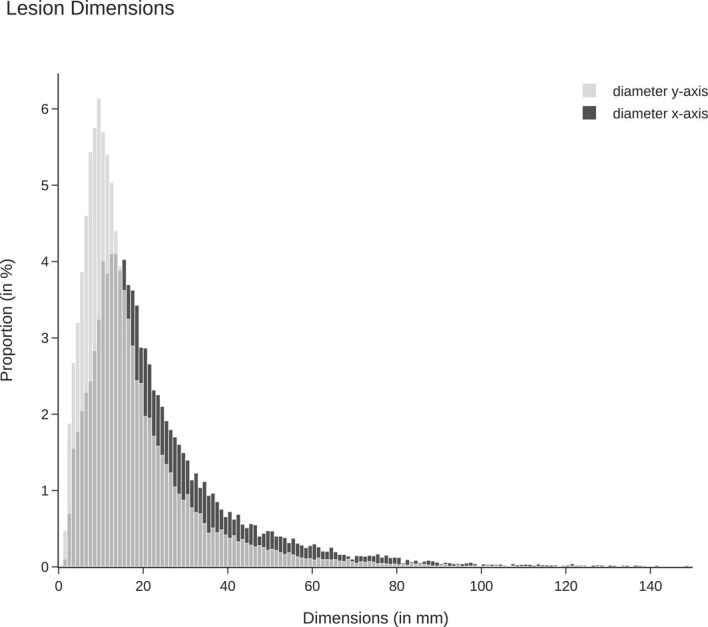
Diameter distribution of DeepLesion. Distribution of diameters in the x and y axes for the DeepLesion training dataset based on RECIST bookmarks identified on key slices. Input dimensions of 50x50x50 mm3 were chosen as they covered 93% and 97% of the distribution in the x and y axes, respectively.

CT scans were resampled using linear interpolation to achieve isotropic voxels with a 1 mm^3^ resolution to address variations in slice thickness and in-plane resolutions across study populations. We extracted patches of 50 × 50 × 50 voxels from the scans centred around a seed point (Extended Data Fig. 7). For the DeepLesion dataset, which provided annotations in the form of RECIST diameters, the seed point was determined by calculating the midpoint of the RECIST diameter. For the other datasets (that is, LUNA16, HarvardRT, LUNG1 and RADIO), which supplied annotations as 3D contours, the seed point was obtained by computing the centre of mass. This approach allows for significantly higher throughput than manual segmentation, which can be more tedious. We then normalized the voxel values in the patches by subtracting −1,024 (lower-bound Hounsfield unit) and dividing by 3,072 (upper-bound Hounsfield unit of 2,048), ensuring the intensity values in the input data ranged between 0 and 1.

### Task-agnostic pretraining of the foundation model

**Extended Data Fig. 8:**
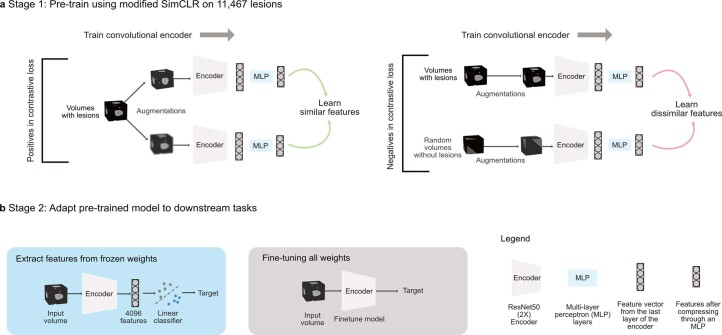
Stages of the implementation pipeline. **a,** We first pre-train using a modified version of the SimCLR on 11.467 lesions. The pre-training process consists of a positive contrastive and a negative contrastive loss component. In the positive contrastive loss, augmentations of the same lesion are made to learn similar features. At the same time, the negative contrastive loss learns different features for volumes with and without lesions. **b**, In the second stage, for each task, different implementation approaches are followed by adapting the pretrained model by either extracting features from a frozen model followed by linearly predicting a target or by fine-tuning all model weights for predicting a target.

**Extended Data Fig. 9:**
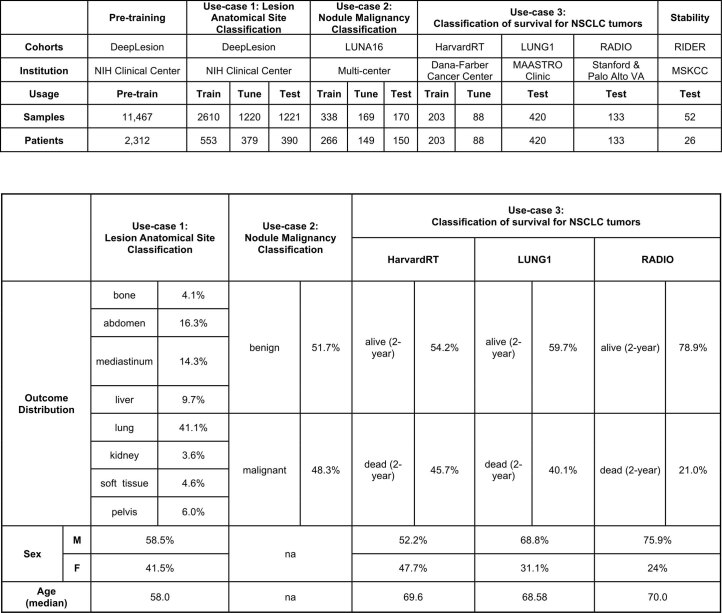
Dataset breakdown. The first table shows the 6 different cohorts used in this study along with eligible scans and patients. A secondary table shows the outcome, sex, and age distribution of each of the cohorts.

We implemented contrastive pretraining using a modified version of the SimCLR framework^5^. The SimCLR framework’s general principle involves transforming a single data sample (for example, a patch taken from a CT scan) into two correlated and augmented samples (for example, the same patch rotated 15° clockwise and flipped horizontally). A convolutional encoder is then used to extract latent representations from these samples. Through a contrastive loss function^44^, the model learns to identify similar representations from the same data sample and dissimilar representations from different data samples (Extended Data Fig. 8). The framework emphasizes effective transformation choices, convolutional encoder architectures and contrastive loss functions for optimal SSL performance. To effectively represent the nature of medical images, we made modifications to each of these components.

Transformations proposed in the original SimCLR framework for natural world images, such as cutout augmentation, Sobel filtering and colour distortion, are unsuited for 3D medical images due to dynamic range and colour depth differences. Therefore, our study applies different augmentations to replace these transformations. For instance, we substituted the random colour jitter transform with a random histogram intensity shift transform, as they both induce variation in intensity distribution.

To extract representations from the transformed 3D volumes, we selected the 3D ResNet50 (ref. ^45^) architecture as our deep convolutional encoder. While the SimCLR authors used a 2D ResNet50 architecture, we opted for its 3D counterpart, which has proven effective in handling 3D medical imaging data^46^.

Regarding loss functions, we extended normalized temperature-scaled cross-entropy loss (NT-Xent)^47^ to support contrastive training for lesion volumes. The modifications include: (1) selecting positive pairs as 3D patches surrounding the lesion’s seed point, (2) choosing negative pairs by randomly sampling 3D patches from the rest of the scan and (3) computing the contrastive loss on these positive and negative pairs, with each iteration comprising *n* positive pairs and *n* × 2(*n* − 1) negative pairs. We also explored different temperature parameters for the NT-Xent loss. However, the original value of 0.1 proposed by the original paper was the most effective.

Our model was pretrained for 100 epochs using an effective batch size of 64 (32 × 2 training nodes) on two NVIDIA Quadro RTX 8,000 graphical processing units (GPUs) taking approximately 5 days. We used stochastic gradient descent as the optimizer, with layer-wise adaptive rate control, momentum and weight-decay enabled. To improve the optimization process, we used learning rate schedulers that combined linear and cosine decay strategies and a warmup phase to modify the learning rate at the beginning of training gradually. While most specifications were consistent with the original SimCLR experiments, we experimented with different batch sizes, patch sizes (50 and 64 mm^3^), learning rates, transforms and model architectures.

We conducted a comparison of our modified SimCLR version with its original form along with various well-known and recent pretraining methods. Before the rise of contrastive approaches, auto-encoder methods were commonly used for pretraining and, therefore, we added this to the comparison. This was implemented using MONAI’s auto-encoder framework, ensuring a parameter count similar to that of ResNet50 (230 million compared to ResNet50’s 200 million). Despite SimCLR’s ongoing popularity^13^, recent methodologies have shown superior results in particular scenarios and tasks. We adapted SwAV^15^ and NNCLR^16^ approaches, combining settings from their original designs with modifications suitable for medical imaging contexts. In our comparative analysis, we maintained uniformity in batch sizes and dataset parameters across all methods, while optimizer and loss-specific settings were aligned with each method’s original configuration.

### Task-specific training of the foundation model

Our foundation model was adapted for a specific task through two approaches: (1) extracting features from the frozen encoder and fitting a linear classifier and (2) transfer learning the pretrained ResNet50 for the given classification task.

We extracted 4,096 features from the foundation model for each data point and used them to train a logistic regression model using the scikit-learn framework^48^. A comprehensive parameter search for the logistic regression model was performed using the optuna hyperparameter optimization framework^49^. No performance improvements were observed through feature selection strategies; therefore, all 4,096 features were used in accordance with linear evaluation strategies prevalent in SSL literature.

Transfer learning through fine-tuning was carried out with all layers updated during training, using cross-entropy loss. A series of randomly chosen augmentations—random flips, random 90° rotations and random translations of ±10 voxels across all axes—were applied throughout the training. Stochastic gradient descent was used for network training, with momentum enabled and step-wise learning rate decay. Following the original SimCLR experiments, configurations and similar parameters (including learning rate, transforms and model architectures) were explored during hyperparameter tuning. Each network was trained for 100 epochs using a single NVIDIA Quadro RTX 8,000 GPU, and the best-performing model checkpoints were chosen on the basis of the tuning set.

For supervised models, we selected four different baselines. First, we randomly initialized the weights of a ResNet50 and trained it using task-specific configurations consistent with fine-tuning the foundation model. Second, the randomly initialized model trained on use case 1 was fine-tuned through transfer learning for use cases 2 and 3. For the third and fourth baselines, publicly available pretrained models were investigated to add comparisons against the state of the art. Specifically, Med3D and Models Genesis were selected on the basis of their relevance to similar domains and tasks, and their established popularity within the community. These models were tailored to each task using configurations that mirrored those of our foundational model, taking into account both their inherent feature representations and transfer learning capabilities.

Task-specific training was conducted on reduced dataset sizes in addition to usic models using these samples with the same configuration as the entire dataset. As the training dataset sizes decreased, we considered training the models for a higher number of epochs; however, models frequently overfitted during extended training. The entire test dataset was used to allow benchmarking across these splits. However, we do not conduct reduced dataset training for use case 3, as it is typical to have inherently small sample sizes in such use cases when compared to task complexity due to study-specific inclusion criteria. Therefore, experiments involving further data reduction in this case do not provide any valuable insights.

### Performance analysis

Validation of the foundation model was performed using several use case-relevant metrics. Lesion anatomical site classification performance was assessed using balanced accuracy as a multi-label counting metric and mAP as a multi-threshold metric. The multi-label metric, balanced accuracy, adjusts class-wise accuracy on the basis of the class distribution at a chosen threshold (0.5). The multi-threshold metric, mAP, enables the examination of a given class’s performance across a range of prediction thresholds. All classes other than the class of interest are considered negatives, and performance is averaged across all possible classes. We avoided using the AUC-receiver operating curve (AUC-ROC) for this use case due to the high proportion of negatives relative to positives, which results in consistently low false-positive rates and might overestimate the AUC. However, due to a more balanced class distribution, nodule malignancy prediction was evaluated using AUC-ROC. NSCLC prognostication networks also used AUC-ROC for evaluation, as it estimates the ranking of participants on the basis of their survival times.

Models underwent pair-wise comparison using permutation tests. *n* permutations (*n* = 1,000) were conducted for each pair, and new models were computed after permuting class labels. Metrics were recalculated after resampling, and a two-sided *P* value was calculated to test the null hypothesis of observations from each pair originating from the same underlying distribution. Additionally, 95% CIs were established for each model using a bootstrap sampling with *n* = 1,000 resamples.

Kaplan–Meier curves were also used to determine the stratification of participants on the basis of their prediction scores for the prognostication models. Groups were selected on the basis of prediction scores on the tuning set, and curves were plotted on the test set for these groups. Multivariate log-rank tests were used to examine the significance of the stratification. Univariate Cox regression models were built using the model predictions as the categorical variables of interest, grouped similarly to the Kaplan–Meier curve.

### Feature visualization and saliency maps

We used the foundation model, top-performing supervised model, Med3D and Models Genesis as feature extractors to obtain 4,096 distinct features (except for Med3D’s 2,048 features) per data point. To enable visual interpretation of these high-dimensional features, we used *t*-stochastic neighbourhood embeddings^50^ at different perplexity values and principal component analysis to reduce their dimensionality to 2D. Points in the 2D visualization were colour-coded according to their respective target classes despite dimensionality reduction being agnostic to these distinctions. Density contours were superimposed over the visualizations to enhance the understanding of group patterns, offering a more comprehensive representation of trends across data points.

To generate saliency maps for each task, the fine-tuned foundation model was used to generate predictions on randomly selected volumes from respective datasets. The fine-tuned foundation model with a single output prediction (corresponding to the predicted target class) was chosen in contrast to the feature extractor as expressing saliency maps over 4,096-dimensional outputs remains challenging in practice. We used a combination of (1) smooth gradient back-propagation, which averages gradients of the output with respect to several noisy inputs, and (2) guided back-propagation, which combines deconvolution with back-propagation, mainly stopping the flow of negative gradients or neurons that decrease the activation signal. The method is termed smooth guided back-propagation^51,52^ and is implemented in the MONAI framework^53^.

### Stability testing

To test the stability of our models, we performed a test–retest and inter-reader variation evaluation. For the test–retest evaluation, we compared model predictions (of outcome) from the best foundation and supervised models generated on chest CT scans taken in a 15-minute interval for 26 patients. ICC was computed using the interrater reliability and agreement package (irr) in R^54^. We also tested the stability of the flattened features computed by the models by calculating Spearman correlation and R^2^.

For the inter-reader variation evaluation, we used the LUNG1 dataset and generated 50 random perturbations sampled from a 3D multivariate normal distribution with zero mean and diagonal covariance matrix for each seed point. Across each dimension, a variance of 16 voxels was used for generating samples. We generated predictions on volumes extracted from perturbed seed points using the best foundation and supervised model, resulting in 50 different prediction sets for each. The mean and variance of the 50 sets were computed for each and compared.

### Biological associations

The GSE103584 dataset contains 130 NSCLC samples that consist of paired CT scans and gene expression profiles generated by RNA sequencing. To analyse gene expression profiles, we filtered them on the basis of cohort mean expression and standard deviation. First, we took only the genes with a higher expression than the overall dataset mean and then picked the top 500 genes on the basis of standard deviation. Next, we performed a correlation analysis comparing the best-supervised and foundation models. To further evaluate foundation model features’ association with tumour biology, we computed the absolute value of the correlation coefficients and performed a gene-set enrichment analysis with all genes with a correlation coefficient above 0.1.

### Reporting summary

Further information on research design is available in the Nature Portfolio Reporting Summary linked to this article.

### Supplementary information

Reporting Summary

## Data Availability

Most of the datasets used in this study are openly accessible for both training and validation purposes and can be obtained from the following sources: (1) DeepLesion^14^, used both for our pretraining and use case 1, (2) LUNA16 (ref. ^55^) used for developing our diagnostic image biomarker, (3) LUNG1 (ref. ^56^) and (4) RADIO^57^ used for the validation of our prognostic image-biomarker model. Imaging and clinical data for the LUNG1 and RADIO datasets were obtained from Imaging Data Commons^58^ collections. The training dataset for our prognostic biomarker model, HarvardRT, is internal to Mass General Brigham institutions and contains sensitive protected health information. Due to privacy concerns and legal restrictions associated with patient data, the complete dataset cannot be made publicly available. However, we have shared the model predictions obtained on this dataset so to ensure that our statistical analyses can be reproduced. Researchers interested in accessing the dataset can submit a formal request detailing the intended use of the data to R.H.M. (RMAK@partners.org). Each request will be evaluated on a case-by-case basis in compliance with the ethical guidelines and agreements under which the data were collected.

## Code availability

The complete pipeline used in this study can be accessed either from the AIM webpage at https://aim.hms.harvard.edu/foundation-cancer-image-biomarker or directly on https://github.com/AIM-Harvard/foundation-cancer-image-biomarker (ref. ^59^). This includes the code for (1) data download and preprocessing: starting from downloading the data to generating train-validation-test splits used in our study; (2) replicating the training and inference of foundation and baseline models across all tasks through easily readable and customizable YAML files (leveraging project-lighter^60^) and (3) code for reproducing our comprehensive performance validation. In addition to sharing reproducible code, we also provide trained model weights, extracted features and outcome predictions for all the models used in our study. Most importantly, we provide our foundation model accessible through a simple pip package install and two lines of code to extract features for your dataset. We also provide a detailed documentation website that can be accessed at https://aim-harvard.github.io/foundation-cancer-image-biomarker/. The final model weights^61^ are made available through the Zenodo platform. The full model implementation is also available through https://mhub.ai/ in a reproducible, containerized, off-the-shelf executable format, allowing fast application in several academic and clinical environments.
