# Supplementary material for: Foundation model for cancer imaging biomarkers
Source: Nat Mach Intell. 2024 Mar 15;6(3):354–67. doi: 10.1038/s42256-024-00807-9 (PMC10957482; doi:10.1038/s42256-024-00807-9)
Supplement: Supplementary file 1 — Reporting Summary [file 42256_2024_807_MOESM1_ESM.pdf]

nature portfolio

Corresponding author(s): Hugo Aerts

Last updated by author(s): 01/23/2024

Reporting Summary

Nature Portfolio wishes to improve the reproducibility of the work that we publish. This form provides structure for consistency and transparency in reporting. For further information on Nature Portfolio policies, see our Editorial Policies and the Editorial Policy Checklist.

Statistics

For all statistical analyses, confirm that the following items are present in the figure legend, table legend, main text, or Methods section.

n/a	Confirmed	
☐	☒	The exact sample size (*n*) for each experimental group/condition, given as a discrete number and unit of measurement
☐	☒	A statement on whether measurements were taken from distinct samples or whether the same sample was measured repeatedly
☐	☒	The statistical test(s) used AND whether they are one- or two-sided<br>*Only common tests should be described solely by name; describe more complex techniques in the Methods section.*
☒	☐	A description of all covariates tested
☒	☐	A description of any assumptions or corrections, such as tests of normality and adjustment for multiple comparisons
☐	☒	A full description of the statistical parameters including central tendency (e.g. means) or other basic estimates (e.g. regression coefficient) AND variation (e.g. standard deviation) or associated estimates of uncertainty (e.g. confidence intervals)
☐	☒	For null hypothesis testing, the test statistic (e.g. *F*, *t*, *r*) with confidence intervals, effect sizes, degrees of freedom and *P* value noted<br>*Give P values as exact values whenever suitable.*
☒	☐	For Bayesian analysis, information on the choice of priors and Markov chain Monte Carlo settings
☒	☐	For hierarchical and complex designs, identification of the appropriate level for tests and full reporting of outcomes
☒	☐	Estimates of effect sizes (e.g. Cohen's *d*, Pearson's *r*), indicating how they were calculated

Our web collection on statistics for biologists contains articles on many of the points above.

Software and code

Policy information about availability of computer code

Data collection	Open-source software: Python 3.8, AcademicTorrents, The Cancer Imaging Archive, Imaging Data Commons, BigQuery<br>Commercial software: Mass General Brigham PACS for HarvardRT
Data analysis	All open source software; Model design and implementation: Python 3.8 and Pytorch 2.0; Online pipeline implementation for model sharing: Python 3.8 and associated packages; Statistical analysis: Python 3.8 and R 3.6.3. All computer code is made available publicly on our Github repository (https://github.com/AIM-Harvard/foundation-cancer-image-biomarker). We provide package management through Python poetry and share a lock file to ensure exact versioning of packages.

For manuscripts utilizing custom algorithms or software that are central to the research but not yet described in published literature, software must be made available to editors and reviewers. We strongly encourage code deposition in a community repository (e.g. GitHub). See the Nature Portfolio guidelines for submitting code & software for further information.

Data

Policy information about availability of data

All manuscripts must include a data availability statement. This statement should provide the following information, where applicable:

- Accession codes, unique identifiers, or web links for publicly available datasets
- A description of any restrictions on data availability
- For clinical datasets or third party data, please ensure that the statement adheres to our policy

The majority of the datasets utilized in this study are openly accessible for both training and validation purposes and can be obtained from the following sources: i) DeepLesion [nihcc.app.box.com/v/DeepLesion] , used both for our pre-training and use-case 1 ii) LUNA16 [luna16.grand-challenge.org] used for developing our diagnostic image biomarker iii) LUNG1 [wiki.cancerimagingarchive.net/display/Public/NSCLC-Radiomics] and iv) RADIO [wiki.cancerimagingarchive.net/display/Public/NSCLC+Radiogenomics] used for the validation of our prognostic image biomarker model. Imaging and clinical data for the LUNG1 and RADIO datasets were obtained from Imaging Data Commons collections. The training dataset for our prognostic biomarker model, HarvardRT, is internal to Mass General Brigham institutions and contains sensitive protected health information. Due to privacy concerns and legal restrictions associated with patient data, the complete dataset cannot be made publicly available. However, we have shared the model predictions obtained on this dataset so to ensure that our statistical analyses can be reproduced. Researchers interested in accessing the dataset can submit a formal request detailing the intended use of the data directed to Raymond H. Mak, M.D., Artificial Intelligence in Medicine (AIM) Program, Mass General Brigham, Harvard Medical School, Harvard Institutes of Medicine – HIM 343, 77 Avenue Louis Pasteur, Boston, MA 02115, P - 617.525.7156, F - 617.582.6037, Email: RMAK@partners.org Each request will be evaluated on a case-by-case basis in compliance with the ethical guidelines and agreements under which the data was collected.

Human research participants

Policy information about studies involving human research participants and Sex and Gender in Research.

Reporting on sex and gender	N/A
Population characteristics	N/A
Recruitment	N/A
Ethics oversight	N/A

Note that full information on the approval of the study protocol must also be provided in the manuscript.

Field-specific reporting

Please select the one below that is the best fit for your research. If you are not sure, read the appropriate sections before making your selection.

☒ Life sciences ☐ Behavioural & social sciences ☐ Ecological, evolutionary & environmental sciences

For a reference copy of the document with all sections, see nature.com/documents/nr-reporting-summary-flat.pdf

Life sciences study design

All studies must disclose on these points even when the disclosure is negative.

Sample size	Sample size was not determined by calculation as this was not a prospective study. Sample size was dependent on availability of data from pre-existing clinical datasets. These datasets were further curated for purposes relevant to the study (see data exclusions below), such sample size was kept as large as possible for purposes of statistical analysis
Data exclusions	Relevant data exclusion criteria was chosen based on the use-case and cohort. For the DeepLesion cohort, CT scans with a slice thickness greater than 3mm were discarded due to insufficient image quality along the z-axis. For the LUNA16 cohort we excluded CT scans where lesions had indeterminate malignancy indicated through consensus (average score) among the radiologists. For HarvardRT, LUNG1 and RADIO we excluded patient scans with missing or corrupt primary tumor annotations (processed using open-source package plastimatch). We also excluded patients with incomplete follow-up information at the two-year time point. Our data download and preprocessing code is end-to-end and explicitly shows all exclusion criteria.
Replication	The software code for the model pipeline and statistical analyses were compiled and cross-checked by members of the research team (not solely the author of the code) to determine if the outputs matched what was reported in the manuscript and figures.
Randomization	Allocation was not random as this study was retrospective.
Blinding	It was not possible to fully blind assessors during data analysis as this was a retrospective study based on pre-existing clinical datasets whereby data curation and data analyses were performed by the same individuals.

Reporting for specific materials, systems and methods

We require information from authors about some types of materials, experimental systems and methods used in many studies. Here, indicate whether each material, system or method listed is relevant to your study. If you are not sure if a list item applies to your research, read the appropriate section before selecting a response.

Materials & experimental systems

n/a	Involved in the study
☒	☐ Antibodies
☒	☐ Eukaryotic cell lines
☒	☐ Palaeontology and archaeology
☒	☐ Animals and other organisms
☐	☒ Clinical data
☒	☐ Dual use research of concern

Methods

n/a	Involved in the study
☒	☐ ChIP-seq
☒	☐ Flow cytometry
☒	☐ MRI-based neuroimaging

Clinical data

Policy information about clinical studies

All manuscripts should comply with the ICMJE guidelines for publication of clinical research and a completed CONSORT checklist must be included with all submissions.

Clinical trial registration	n/a - this study was not a clinical trial
Study protocol	This was not a clinical trial but a retrospective study using pre-existing clinical datasets. The study protocols are described in the manuscript and can be found online for the public datasets.
Data collection	Clinical data was collected a priori to the study under separate protocols. DeepLesion, LUNA16, LUNG1 and RADIO datasets are publicly available and have been previously used in several studies (Refer to details and citation in the manuscript for each of these datasets). HarvardRT, our internal dataset, is a cohort of 317 patients with stage I-IIIB NSCLC treated with radiation therapy at the Dana-Farber Cancer Institute and Brigham and Women's Hospital, Boston, MA, US, between 2001 and 2015. This dataset has also been used in a previous study from our group and is cited in the manuscript.
Outcomes	This study looked at different outcomes depending on the clinical use-case of interest. Our first, technical validation use-case focused on predicting anatomical site of the lesion from one of 8 anatomical sites. This was evaluated using balanced accuracy calculated across the sites and mean Average Precision (mAP). For the use-case of nodule malignancy prediction, we determined the likelihood of a nodule to be malignant and compared performance to radiologist labels using AUC-ROC and mAP. Finally, in the case of NSCLC prognostication, we chose to predict two-year overall survival as the endpoint, as this was the most stringent and most clinically relevant outcome measure for purposes of assessing prognostic power of the model on a clinical population of cancer patients. We evaluated our predicted survival outcome using 1) ROC-AUC when compared with the true survival outcome, 2) Kaplan-Meier curves to determine the ability of our predicted score to stratify patient groups, and 3) Univariate cox regression to demonstrate the prognostic power of our compared models.

March 2021
